# Editorial: Herbal medical products for metabolic diseases - new integrated pharmacological approaches

**DOI:** 10.3389/fphar.2024.1464176

**Published:** 2024-08-09

**Authors:** Antony Stalin, Abd El-Latif Hesham, Avdesh Mishra, Quan Zou, Savarimuthu Ignacimuthu

**Affiliations:** ^1^ Institute of Fundamental and Frontier Sciences, University of Electronic Science and Technology of China, Chengdu, China; ^2^ Genetics Department, Faculty of Agriculture, Beni-Suef University, Beni-Suef, Egypt; ^3^ Department of Electrical Engineering and Computer Science, Texas A&M University Kingsville, Kingsville, TX, United States; ^4^ Xavier Research Foundation, St. Xavier’s College, Palayamkottai, Tamil Nadu, India

**Keywords:** metabolic diseases, natural compounds, artificial intelligence, bioinformatics (computational biopharmaceutics and modeling), pharmaceutical chemistry, network pharmacology

Type 2 diabetes mellitus (T2DM), obesity, non-alcoholic fatty liver disease (NAFLD), metabolic dysfunction-associated fatty liver disease (MAFLD) and metabolic dysfunction-associated steatotic liver disease (MASLD) are interrelated metabolic disorders that significantly affect global health (Younossi et al., 2019; Chan et al., 2023). These diseases often lead to serious complications, including cancer and diabetic vascular problems, and are closely linked to autophagic activity, mitophagy and ferroptosis. Insulin resistance and other metabolic abnormalities play a central role in these diseases and contribute to their progression and severity. In particular, these metabolic abnormalities are major causes of hepatocellular carcinoma (HCC), highlighting the urgent need for effective interventions (Wang Y. et al., 2023).

Recent research has emphasized the potential of traditional medicines, plant extracts and natural substances for the treatment of these diseases. Of particular note is traditional Chinese medicine (TCM), which offers a variety of treatments that target underlying metabolic disorders (Hwang et al., 2022). Studies have shown that TCM can modulate autophagic activity, promote mitophagy and reduce ferroptosis, thereby alleviating insulin resistance and other metabolic abnormalities (Undamatla et al., 2023; Xie et al., 2023). These findings suggest that the integration of natural products and traditional medicine with modern therapeutic approaches may open new avenues for the treatment of T2DM, obesity, NAFLD/MAFLD and their associated complications, including HCC (Bai et al., 2020; Wang X. et al., 2023). As research progresses, the role of natural agents and traditional medicinal practices in the treatment and potential alleviation of these widespread health problems may become increasingly important.

In the Research Topic *“Herbal Medical Products for Metabolic Diseases - New Integrated Pharmacological Approaches”*, 11 articles were published, mainly focusing on plant metabolites, including TCM, for the treatment of metabolic diseases.

Ji-Ni-De-Xie (JNDX), a traditional botanical drug preparation from China used in Tibetan medicine for the treatment of T2DM, was studied by Tao et al. to understand its underlying mechanisms. JNDX significantly decreased fasting blood glucose, serum glycosylated protein, insulin resistance, inflammatory cytokines, triglycerides, total cholesterol and low-density lipoprotein cholesterol while increasing insulin sensitivity and high-density lipoprotein cholesterol in T2DM rats. Metagenomic analyses revealed that JNDX improved gut microbiota dysbiosis, specifically by altering bacteria associated with bile acid metabolism. JNDX also corrected disorders of bile acid metabolism by increasing cholic acid levels and decreasing ursodeoxycholic acid levels, and it improved FXR and FGF15 protein and mRNA expression while inhibiting CYP7A1 expression in the liver. This study revealed that JNDX effectively improved insulin resistance, hyperglycemia, hyperlipidemia and inflammation in T2DM rats through its regulation of bile acid metabolism and activation of the FXR/FGF15 pathway.

Similarly, Xia et al. summarized in their review article that botanical drug remedies show promise in combating insulin resistance, with anthraquinone extracts garnering attention for their role in improving insulin sensitivity and treating diabetes. The findings discussed in this review suggest that anthraquinones represent a promising therapeutic strategy for combating insulin resistance and associated metabolic diseases.

Hyperlipidemia is associated with obesity and is a serious health problem for many people. Xiao et al. reported that TCM Yinlan Tiaozhi capsule (YL) significantly reduced the levels of TC, TG, LDL-C, ll6, Tnf-a and Vegfa in mice with Triton WR-1339-induced hyperlipidemia and significantly increased the levels of HDL-C and Alb. The reduction in serum lipids was related to the alteration of metabolic abnormalities and maintenance of the dynamic balance of metabolites.

NAFLD, also known as MAFLD or MASLD, is a global health problem because it is associated with obesity, insulin resistance and other metabolic abnormalities and is also the leading cause of HCC. Methylsulfonylmethane (MSM), an organic sulfur compound found in various plants and animals, exerts antioxidant and anti-inflammatory effects. Han et al. highlighted their ability to assess the anti-obesity activity and autophagy-related mechanisms of MSM and suggested that MSM ameliorates hepatic steatosis by enhancing autophagic flux via an AMPK/mTOR/ULK1-dependent signaling pathway.

In addition, Wang et al. comprehensively discussed recent advances in the possible mechanisms of pathogenesis and progression of MASLD-related HCC in their review article. They also discussed the application of various bioactive metabolites to attenuate MASLD-related HCC through different modulatory mechanisms involving anti-inflammatory, lipid metabolic and microbial pathways in the gut, providing valuable information for the future treatment and prevention of MASLD-related HCC. They also discussed recent findings suggesting that ferroptosis, a form of regulated cell death, plays a role in the progression from MASLD to HCC and is a potential therapeutic target.

Gao et al. reviewed the relationship between diabetic vascular complications and autophagic activity, a process important for cellular homeostasis that involves lysosomal degradation of protein aggregates, damaged organelles and pathogens. Dysregulated autophagy has been associated with vascular abnormalities in both microvessels and large vessels in diabetes. They also discussed the potential of TCMs, with their ability to target multiple metabolic pathways, which offers promising prospects as treatment strategies, especially given the complex etiology of diabetic vascular lesions. This complexity often renders single-target treatments ineffective, whereas TCMs can address multiple autophagic targets simultaneously.

Similarly, Zhang et al. provided an overview of advances in medicinal plants and their active metabolites for the treatment of ischemia‒reperfusion injury in stroke, a common and severe neurological disorder that includes ischemic (85% of cases) and hemorrhagic (15% of cases) forms. This review describes in detail how mitophagy and ferroptosis contribute to the pathogenesis of stroke and discusses the potential of medicinal plants to act on these processes, offering new insights for drug development.

The increase in chronic alcohol consumption has become a major global health problem and leads to a number of liver diseases, including steatosis, steatohepatitis, cirrhosis and HCC. Excessive alcohol consumption often causes hangovers and inflammatory liver damage, and current treatment options are inadequate. In their study, Yang et al. investigated the effects of psyllium fiber (PF), known for its gastrointestinal benefits, on hangover symptoms. Excessive alcohol consumption was found to activate alcohol-metabolizing enzymes in the small intestine and liver, leading to inflammatory damage and increased alcohol metabolites such as acetaldehyde and acetone, which correlate with hangover symptoms in mice. Compared with control mice, PF-treated mice exhibited significant improvement in hangover symptoms and reduced hepatic inflammation. *In vitro* experiments with HepG2 cell lines and semipermeable membranes have shown that PF inhibits alcohol absorption.

Shizao decoction (SZD) is a TCM that has a therapeutic effect on cirrhotic ascites (CAS). In their study, Li et al. developed a new integrated strategy involving network analysis combined with pharmacokinetics and metabolomics to investigate the main targets and mechanisms of SZD in the treatment of CAS. The results showed that SCD can reduce liver tissue inflammation, inhibit collagen fiber hyperplasia and improve liver function.

Fazmiya et al. investigated the efficacy of *Acacia arabica* (Lam.) Willd. and *Cinnamomum camphora* (L.) J. Presl. Vaginal suppositories for heavy menstrual bleeding (HMB) and their effects on participants' health-related quality of life (HRQoL) were analyzed using machine learning algorithms, and the results revealed that the use of a vaginal suppository is effective, inexpensive and safe for controlling HMB.

Ariyanto provided an overview of the efficacy of botanical drugs in the treatment of metabolic diseases through epigenetic modifications in his review paper to provide insight into the research and development strategies for botanical drugs as pharmacotherapies for metabolic diseases.

In summary, this Research Topic highlights the key interest as well as the potential of traditional medicine, especially TCM and other botanical drugs, in modulating metabolic pathways and addressing metabolic disorders. It also discusses the role of autophagy, mitophagy and ferroptosis in the development and treatment of metabolic diseases. In addition, the integration of natural products and traditional medicine with modern therapeutic approaches for comprehensive treatment strategies has been addressed.

Moreover, some areas are not covered, such as detailed research on specific molecular mechanisms and pathways involved in all plant metabolites, comparative studies between botanical drugs and modern pharmacological treatments, long-term clinical trials and safety profiles, standardized formulations, dosages and quality control of botanical drugs, *etc.*, Overall, this Research Topic highlights the recent advances in botanical drugs for various interrelated metabolic diseases such as T2DM, obesity, cancer and NAFLD and emphasizes the importance of further research and integration with modern medicine.

## References

[B1] Bai Q. Y. Tao S. M. Tian J. H. Cao C. R. (2020). Progress of research on effect and mechanism of Scutellariae Radix on preventing liver diseases. Zhongguo Zhong Yao Za Zhi 45, 2808–2816. 10.19540/j.cnki.cjcmm.20200224.403 32627454

[B2] Chan W. K. Chuah K. H. Rajaram R. B. Lim L. L. Ratnasingam J. Vethakkan S. R. (2023). Metabolic dysfunction-associated steatotic liver disease (MASLD): a state-of-the-art review. J. Obes. Metab. Syndr. 32, 197–213. 10.7570/jomes23052 37700494 PMC10583766

[B3] Hwang K. A. Hwang Y. Hwang H. J. Park N. (2022). Hepatoprotective effects of radish (raphanus sativus L.) on acetaminophen-induced liver damage via inhibiting oxidative stress and apoptosis. Nutrients 14, 5082. 10.3390/nu14235082 36501112 PMC9737327

[B4] Undamatla R. Fagunloye O. G. Chen J. Edmunds L. R. Murali A. Mills A. (2023). Reduced mitophagy is an early feature of NAFLD and liver-specific PARKIN knockout hastens the onset of steatosis, inflammation and fibrosis. Sci. Rep. 13, 7575. 10.1038/s41598-023-34710-x 37165006 PMC10172344

[B5] Wang X. Liu B. Liu Y. Wang Y. Wang Z. Song Y. (2023a). Antioxidants ameliorate oxidative stress in alcoholic liver injury by modulating lipid metabolism and phospholipid homeostasis. Lipids 58, 229–240. 10.1002/lipd.12377 37547958

[B6] Wang Y. Fleishman J. S. Li T. Li Y. Ren Z. Chen J. (2023b). Pharmacological therapy of metabolic dysfunction-associated steatotic liver disease-driven hepatocellular carcinoma. Front. Pharmacol. 14, 1336216. 10.3389/fphar.2023.1336216 38313077 PMC10834746

[B7] Xie D. Li K. Feng R. Xiao M. Sheng Z. Xie Y. (2023). Ferroptosis and traditional Chinese medicine for type 2 diabetes mellitus. Diabetes Metab. Syndr. Obes. 16, 1915–1930. 10.2147/DMSO.S412747 37398945 PMC10312342

[B8] Younossi Z. M. Golabi P. De Avila L. Paik J. M. Srishord M. Fukui N. (2019). The global epidemiology of NAFLD and NASH in patients with type 2 diabetes: a systematic review and meta-analysis. J. Hepatol. 71, 793–801. 10.1016/j.jhep.2019.06.021 31279902

